# Knowledge, attitudes, practices, and adherence to multiple micronutrient supplementation among pregnant women in Rwanda: a mixed-methods study

**DOI:** 10.3389/fnut.2026.1874824

**Published:** 2026-08-28

**Authors:** Eric Matsiko, Annet Birungi, Eliphaz Tuyisenge, Patrick Izabayo Rudatinya, Phionah Nziza, Jean de Dieu Habimana, Harriet Gyamfuah Adu-Amoah, Samson Desie

**Affiliations:** 1 School of Public Health, College of Medicine and Health Sciences, University of Rwanda, Kigali, Rwanda; 2 UNICEF Rwanda, Kigali, Rwanda

**Keywords:** antenatal care, knowledge, attitudes, and practices (KAP), MMS, multiple micronutrient supplementation, supplement adherence

## Abstract

**Introduction:**

Micronutrient deficiencies among pregnant women remain a public health concern in Rwanda. Multiple Micronutrient Supplementation (MMS) was introduced through routine antenatal care services to address this challenge. However, evidence on women's knowledge, attitudes, practices, and adherence to MMS is limited. This study assessed knowledge, attitudes, practices, and adherence to MMS among pregnant and lactating women and examined factors associated with knowledge, attitudes, and adherence.

**Methods:**

A mixed-methods study was conducted in December 2024 in the Rwandan districts of Gasabo, Rutsiro, and Burera, where the MMS programme was implemented. Quantitative data were collected from 484 pregnant and lactating women using a structured questionnaire. Qualitative data were gathered through 18 focus group discussions, 12 exit interviews, and key informant interviews with healthcare providers and district officials. Quantitative data were analyzed using Stata, while qualitative data were analyzed thematically.

**Results:**

Although adherence to MMS was high (92%), only 26.7% of participants demonstrated high knowledge, while 56.6% reported positive attitudes toward MMS. No socio-demographic factors were independently associated with high MMS knowledge. Positive attitudes were significantly associated with high knowledge (AOR = 5.10, 95% CI: 3.09–8.43), being a lactating mother (AOR = 2.30, 95% CI: 1.37–3.86), and employment, whereas women living farther from health facilities were less likely to report positive attitudes (AOR = 0.58, 95% CI: 0.36–0.93). Positive attitudes were the only independent predictor of adherence (AOR = 3.29, 95% CI: 1.42–7.61). Qualitative findings showed that perceived benefits, trust in healthcare providers, family support, and community follow-up facilitated adherence, whereas misconceptions, side effects, and limitations in counseling hindered optimal MMS use.

**Conclusions:**

High self-reported adherence to MMS was achieved despite limited knowledge, suggesting that positive attitudes, perceived benefits, and trust in healthcare providers are key drivers of adherence. Strengthening ANC counseling, addressing misconceptions, and promoting early ANC attendance may support the effective implementation and scale-up of MMS programmes in Rwanda and similar settings.

## 1 Introduction

Micronutrient deficiencies during pregnancy remain a significant public health concern, particularly in low- and middle-income countries (LMICs). Pregnant women are especially vulnerable to deficiencies in essential micronutrients, including iron, folate, iodine, zinc, vitamins A and D, riboflavin, and vitamins B6 and B12 (1). Inadequate intake of these nutrients during pregnancy can adversely affect both maternal health and pregnancy outcomes (2–4). The increased metabolic demands associated with pregnancy, coupled with poor dietary quality, often result in unmet nutritional needs, leading to heightened risks of complications such as preterm birth, small-for-gestational-age (SGA) infants, neural tube defects (NTDs), and various maternal health conditions (5–8).

In Rwanda, micronutrient malnutrition is prevalent among pregnant women. Data from the Rwanda Demographic and Health Survey (RDHS) Supplemental Report on Micronutrients (9) underscore the magnitude of this issue. Anemia affects 17.6% of pregnant women, and 15% are iron-deficient. Additionally, 28% of pregnant women experience vitamin A insufficiency, while vitamin B12 deficiency affects 43.5%, and the risk of deficiency is present in 72% of women. Given the high burden of multiple micronutrient deficiencies among pregnant women, interventions targeting iron deficiency alone are unlikely to fully address maternal nutritional needs. Although Rwanda has implemented an iron-folic acid supplementation programme, its coverage remains low (9) and it does not address deficiencies in other essential micronutrients (5). These gaps provide a strong rationale for introducing MMS as a more comprehensive antenatal nutrition intervention. Moreover, the IFA program primarily addresses iron deficiency anemia, leaving other micronutrient deficiencies unaddressed. Given the multifaceted impact of micronutrient deficiencies during pregnancy, MMS has emerged as a promising public health intervention. MMS not only offers comparable benefits to iron-folate supplementation in reducing anemia but also addresses other micronutrient deficiencies and supports fetal growth, neonatal health, and infant development (7, 10, 11). MMS represents a safe and effective strategy to bridge nutritional gaps and fulfill the elevated micronutrient demands of pregnancy, particularly in settings where dietary diversity is limited (8).

Cost-effective analyses further support the adoption of MMS, indicating that it offers substantial economic benefits relative to traditional IFA supplementation (12). Evidence suggests that MMS reduces maternal anemia, preterm birth, SGA births, and the incidence of NTDs in newborns (7, 8, 11). By ensuring sufficient intake of key nutrients, including iron, folic acid, docosahexaenoic acid (DHA), and vitamin D, MMS not only promotes maternal wellbeing and metabolic health but also optimizes placental function (13). For the fetus, MMS plays a critical role in promoting brain development, improving neurocognitive function, and mitigating the risk of neurodevelopmental disorders (14). It is particularly advantageous in high-risk pregnancies, where it has been associated with better outcomes in conditions such as pre-eclampsia and intrauterine growth restriction (IUGR) (11).

The World Health Organization (WHO) now recommends the use of MMS as part of comprehensive nutritional interventions to improve maternal micronutrient status and pregnancy outcomes (5). The updated WHO guidelines create a valuable opportunity for countries to incorporate MMS into national health strategies, thereby enhancing the provision of quality nutrition services through antenatal care (ANC) platforms. In line with this WHO recommendation and recognizing the potential benefits of MMS, Rwanda initiated the integration of MMS into ANC services in 2024. This effort commenced with a pilot program launched in seven districts in 2024, with plans for expansion to other districts in 2025. As part of the scale-up preparations, UNICEF Rwanda, in collaboration with the Rwanda Biomedical Center and the Ministry of Health, commissioned an assessment to evaluate knowledge, attitudes, and practices (KAP) related to MMS in the context of Rwanda.

Although MMS has demonstrated effectiveness in improving maternal and birth outcomes, evidence on the behavioral and implementation factors influencing adherence remains limited in Rwanda. Understanding how women perceive MMS, the barriers and facilitators they encounter, and the role of family and health system support is particularly important during the early stages of program implementation. Such evidence is critical for informing the successful scale-up of MMS and ensuring that the benefits demonstrated in clinical studies are translated into routine practice.

The current study was guided by the Health Belief Model (HBM), which proposes that health behaviors are influenced by perceived benefits, perceived barriers, cues to action, and self-efficacy (15). In the context of MMS, women's knowledge, perceptions of the benefits and risks of supplementation, support from family members and healthcare providers, and confidence in adhering to supplementation may influence continued use. Previous studies have shown that greater awareness of the benefits of maternal nutritional supplementation is associated with improved adherence (16–18). Guided by this framework, the study assessed knowledge, attitudes, and practices related to MMS among pregnant and lactating women and identified factors associated with adherence. By examining behavioral and implementation determinants of MMS use during the early phase of implementation, the study provides context-specific evidence to inform Rwanda's national MMS scale-up and support strategies that improve adherence.

## 2 Materials and methods

### 2.1 Design and setting

This study employed a convergent mixed-methods design, in which quantitative and qualitative data were collected concurrently during the same study period, analyzed separately, and integrated during interpretation through triangulation. The quantitative component assessed knowledge, attitudes, practices, and adherence to MMS among pregnant and lactating women and identified factors associated with these outcomes. The qualitative component explored participants' perceptions, experiences, barriers, and facilitators related to MMS uptake and adherence among women, husbands, community health workers, healthcare providers, and district-level health officials. The two components were analyzed independently, and the qualitative findings were used to explain, contextualize, and enrich the quantitative results, providing a more comprehensive understanding of the behavioral, social, and health-system factors influencing MMS implementation.

The qualitative component comprised key informant interviews (KIIs) and focus group discussions (FGDs). Participants included pregnant women attending ANC who were receiving MMS, lactating mothers with infants aged 3 months or younger who had used MMS during pregnancy, husbands, community health workers, health facility staff providing ANC services (nurses and midwives), and district health directors. Data collection for both the quantitative and qualitative components was conducted concurrently in December 2024 in the districts of Gasabo, Rutsiro, and Burera, where the MMS programme was being implemented.

### 2.2 Sampling and sample size

We randomly selected a representative sample of 12 health centers from a total of 52 health centers across the three districts, allocating four health centers to each district. From a comprehensive, alphabetically arranged list of all health centers in each district, we used the random function in Excel for selection. Within each selected health center catchment area, four villages were purposively selected to ensure representation of both communities located near the health facility and those situated farther away, thereby capturing variation in access to services. We applied random sampling to select participants from a comprehensive list of pregnant women enrolled in the MMS program, as well as lactating mothers who had used MMS in the selected villages. We obtained the complete lists from the respective health centers.

We targeted a minimum sample size of 484 pregnant women and lactating mothers, calculated using Yamane Taro's formula, with a 95% confidence level, 80% power, and a 5% margin of error. We based the calculation on a total population of 28,889 pregnant women enrolled in the MMS program across the three districts as of October 2024. We proportionally allocated the sample across the three districts according to the number of pregnant women receiving MMS in each district.

For the qualitative component, participants were selected using purposive sampling to ensure representation of key stakeholders involved in the implementation and use of MMS across the three implementation districts. Eligible participants included pregnant women attending ANC who were receiving MMS, lactating mothers with infants aged 3 months or younger who had used MMS during pregnancy, husbands, community health, nurses and midwives providing ANC services, and district health directors overseeing maternal and child health programmes.

Pregnant and lactating women were identified with the support of healthcare providers at selected health centers. Husbands were recruited through participating women, while community health workers, healthcare providers, and district health directors were selected based on their roles in MMS implementation. We conducted 18 FGDs, comprising seven with women, five with husbands, and six with community health workers, with approximately eight participants per group. In addition, 12 exit interviews were conducted with women immediately after ANC visits to obtain real-time feedback on MMS service delivery, and key informant interviews were conducted with three health facility staff responsible for ANC (nurses or midwives) and district health directors. The number of FGDs was determined purposively to ensure representation across stakeholder groups, while data collection continued until thematic saturation was achieved, with no substantially new themes emerging.

### 2.3 Data collection procedures

The quantitative questionnaire and qualitative interview guides were developed based on the study objectives, existing literature on micronutrient supplementation, and consultation with maternal nutrition experts from UNICEF Rwanda, the Rwanda Biomedical Center, and the Ministry of Health. The tools were reviewed for content validity, translated into Kinyarwanda, and back-checked for consistency. A pilot test was conducted in a non-study health facility, and minor revisions were made to improve clarity, wording, and sequencing of questions before implementation. Enumerators administered a quantitative questionnaire, developed specifically for this study and programmed into the Kobo Collect application for data collection. They submitted completed questionnaires daily through the application, and the data analyst reviewed them for completeness and accuracy. Moderators and note-takers facilitated the FGDs and audio-recorded them with participants' consent. Similarly, experienced facilitators conducted the KIIs using a translated interview guide and audio-recorded the sessions upon receiving consent. Additionally, they conducted 12 exit interviews with pregnant women immediately after their ANC visits to capture real-time feedback on the MMS service.

### 2.4 Data analysis

Knowledge and attitude scores were computed by summing responses to relevant questionnaire items, as described in Supplementary material 1. The internal consistency of the knowledge and attitude scales was assessed using Cronbach's alpha. The 12-item knowledge and attitude scales showed acceptable internal consistency, with Cronbach's alpha coefficients of 0.60 and 0.65, respectively. Because no validated cut-off exists for knowledge and attitude scores related to multiple micronutrient supplementation, participants scoring at least 50% of the maximum attainable score were categorized as having high knowledge or positive attitudes, while those scoring below 50% were categorized as having low or moderate knowledge and attitudes. This predefined 50% threshold was adopted as a pragmatic criterion to distinguish participants demonstrating at least half of the expected knowledge or favorable attitudes from those with lower scores, consistent with approaches used in KAP studies where no validated scoring system is available (19). Adherence was defined as self-reported consistent use of MMS according to recommended guidelines and was categorized as adherent or non-adherent.

Bivariate analyses were conducted using Pearson's chi-square tests to examine associations between independent variables and the outcome variables (knowledge, attitudes, and adherence). Variables with a *p-value* < 0.20 in bivariate analyses were included in multivariable logistic regression models to identify factors independently associated with high knowledge, positive attitudes, and adherence to MMS. Adjusted odds ratios (AORs) with 95% confidence intervals (CIs) were reported, and statistical significance was determined at *p* < 0.05. Multicollinearity among independent variables was assessed using the Variance Inflation Factor (VIF). All VIF values were below 2, indicating no evidence of problematic multicollinearity.

Qualitative data were analyzed using thematic analysis supported by ATLAS.ti. Audio recordings from the FGDs, KIIs, and exit interviews were transcribed verbatim and translated from Kinyarwanda into English. Transcripts were reviewed for accuracy and familiarization before coding. A coding framework was developed using deductive codes informed by the study objectives and key constructs of the Health Belief Model, while allowing additional inductive codes to emerge from the data. Related codes were grouped into categories and broader themes, including knowledge, attitudes, adherence, perceived benefits, perceived barriers, facilitators, and health-system influences on MMS use. Coding decisions and emerging themes were discussed among members of the research team and refined iteratively to ensure consistency and alignment with the data. The trustworthiness of the qualitative findings was ensured through triangulation of multiple stakeholder groups and data collection methods, standardized and pilot-tested interview guides, collaborative coding and iterative refinement of themes, and detailed descriptions of the study setting, participants, and sampling procedures. Qualitative findings were then integrated with the quantitative results during interpretation to explain, contextualize, and enrich the statistical findings.

### 2.5 Ethical considerations

We obtained ethical approval from the Rwanda National Ethics Committee (RNEC 638/2024) and secured written informed consent from all participants. Before participation, we explained the study's purpose, voluntary nature, potential risks and benefits, and confidentiality measures.

## 3 Results

### 3.1 Participants Profile

A total of 484 women participated in the study, comprising pregnant women in their first and second trimesters, pregnant women in their third trimester, and lactating mothers. The study population was relatively young, with approximately two-thirds aged 30 years or younger. Most participants were living with a partner, had attained at least primary education, and were able to read and write. Nearly half were engaged in business or salaried employment, while the remainder were involved in agriculture or reported having no occupation (Table 1).

**Table 1:** Participants' profile.

Characteristics	Count *n* = 484	%
Pregnancy status		
1st and 2nd trimesters	213	44.0
3rd trimester	163	33.7
Lactating mothers	108	22.3
Age		
≤ 30	224	67.0
≥ 30	160	33.0
Marital status		
Living with no partner	58	12.0
Living with a partner	426	88.0
Education		
None	44	9.0
Any Primary	240	49.6
Any secondary and higher	200	41.4
Ability to read and write		
No	42	8.7
Yes	442	91.3
Occupation		
Agriculture	125	25.8
Business/salaried	234	48.4
No work	125	25.8
Religion		
Catholic	154	32.0
Protestant/Seventh-day Adventist/Muslim	329	68.0
Walking time to health facility		
≤ 30 min	205	42.4
>30 to 60 min	142	29.3
≥60 min	137	28.3

The sample was diverse in terms of religious affiliation, although Protestant, Seventh-day Adventist, and Muslim participants collectively constituted the majority. Access to health services varied, with less than half of the participants (42.4%) reporting a travel time of at least 30 min to the nearest health facility (Table 1).

### 3.2 Knowledge of multiple micronutrient supplementation

**Figure 1:**
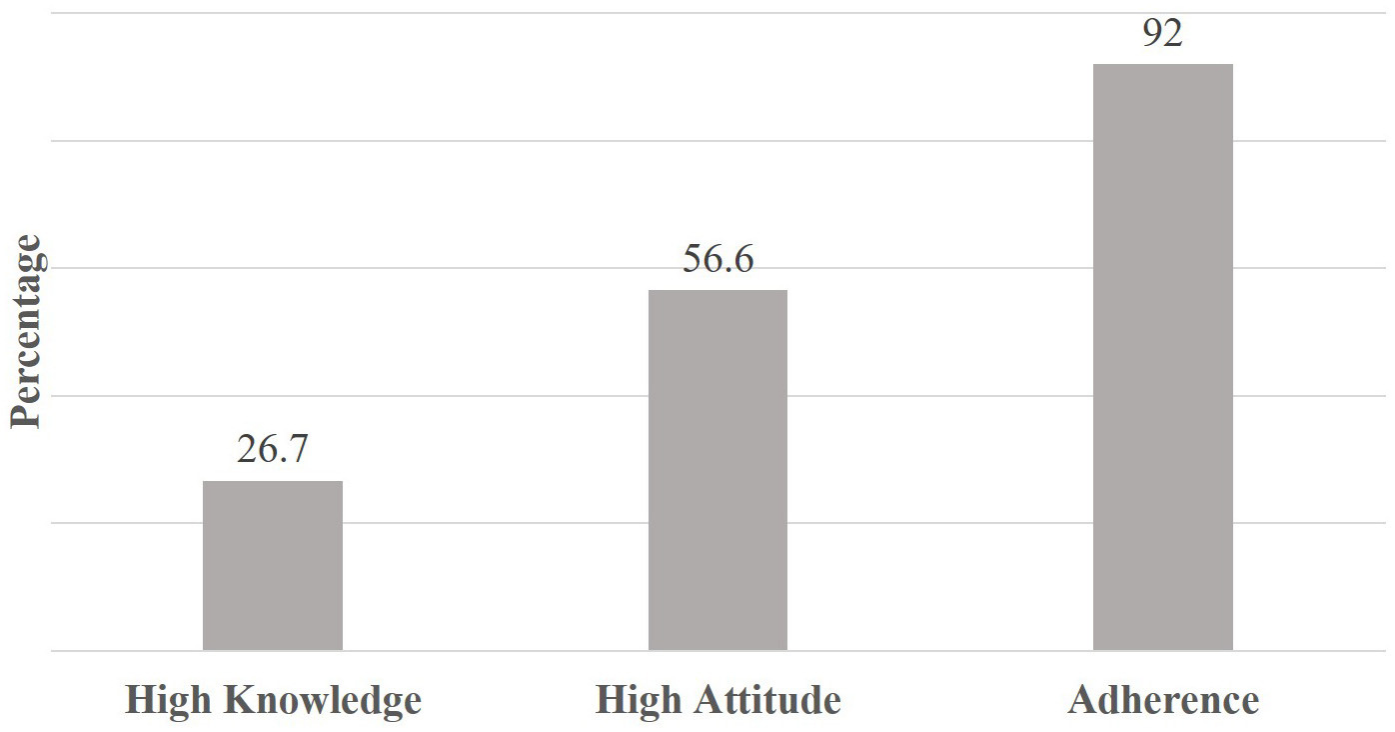
Percentage of study participants with high knowledge, positive attitudes, and adherence to multiple micronutrient supplementation.

Overall, 26.7% of participants demonstrated high knowledge of MMS (Figure 1) according to the scoring procedure described in Supplementary material 2, and Table 2 presents the bivariate and multivariable analyses of factors associated with high MMS knowledge. In the bivariate analysis, marital status was significantly associated with knowledge levels. However, none of the variables remained significantly associated with high MMS knowledge in the adjusted model. The qualitative findings support this interpretation. Healthcare providers and community health workers were consistently identified as the primary sources of MMS information, regardless of participants' age, education level, marital status, or place of residence. Women reported receiving information through antenatal care services, community outreach activities, and interactions with community health workers. As one nurse from Rutsiro explained, “*we focus on explaining their benefits. We go into great detail to ensure they understand”* (Nurse, Rutsiro District). The broad dissemination of information through routine maternal health services may have contributed to the relatively similar knowledge levels observed across socio-demographic groups.

**Table 2:** Factors associated with high knowledge of multiple micronutrient supplementation among pregnant and lactating women in Rwanda.

Characteristics	High knowledge *n* (%)	*p-value*	AOR	95% CI	*p-value*
Pregnancy status					
1st and 2nd trimester	57 (26.7)	0.892			
3rd trimester	45 (26.7)				
Lactating mothers	27 (25.0)				
Age					
≤ 30	79 (24.4)	0.108	1		
≥ 30	50 (31.2)		1.37	0.86–2.17	0.181
Marital status					
Living with a partner	60 (23.0)	0.049	1		
Living with no partner	69 (31.0)		1.40	0.91–2.18	0.124
Education					
None	11 (25.0)	0.078	1		
Any primary	54 (22.5)		0.86	0.40–1.83	0.698
Any secondary and higher	64 (32.0)		1.54	0.71–3.30	0.267
Occupation					
Agriculture	31 (24.8)	0.635			
Business/casual labor	67 (28.6)				
No work	31 (42.8)				
Religion					
Catholic	82 (30.5)	0.195	1.00		
Protestant and others	82 (25.0)		1.42	0.92–2.20	0.099
Walking time to the nearest health center					
≤ 30 min	50 (24.4)	0.270			
>30 to 60 min	45 (31.7)				
≥60 min	34 (24.8)				

AOR, Adjusted Odds Ratio; CI, Confidence Interval. Variables with *p* < 0.20 in bivariate analysis were included in the multivariable logistic regression model.

Many women demonstrated a general awareness of the benefits of MMS but lacked detailed knowledge of its composition and specific functions. A pregnant woman from Burera District explained, “*I know they help the baby, but I do not know exactly what is inside them”* (Pregnant Woman, Burera District). While women acknowledged the benefits of MMS, gaps in understanding remained. Some women were uncertain about the composition of MMS and whether it differed from supplements they had previously received during pregnancy. Misconceptions regarding the contents and appearance of MMS were also evident. One lactating mother explained, “*When they gave me the tablets, I wondered if they also included a deworming component. Previously, they gave us red tablets and a deworming medication. Do these tablets contain a deworming component? That is something I have been curious about”* (Lactating Mother, Gasabo District). Others questioned whether MMS had simply replaced previous supplements without understanding the differences between them. A participant asked, “*I wanted to know if these tablets replaced the previous ones”* (Lactating Mother, Gasabo District). These findings suggest that although women were generally aware of the benefits of MMS, their understanding of its composition and distinction from other medications and supplements remained limited.

Healthcare providers attributed these knowledge gaps to limitations in counseling. High patient volumes, limited consultation time, and reliance on group education sessions often constrained opportunities for individualized discussions. A district health official from Rutsiro observed that “*a mother who comes to the health facility for antenatal care often has limited time, so the counseling she receives is brief”* (District Health Official, Rutsiro District). Providers also highlighted gaps in MMS-specific training and educational materials.

### 3.3 Attitudes toward multiple micronutrient supplementation

As shown in Figure 1, overall, 56.6% of participants demonstrated positive attitudes toward MMS according to the scoring procedure described in Supplementary material 2. Table 3 presents the bivariate and multivariable analyses of factors associated with positive attitudes toward MMS. In the bivariate analysis, pregnancy status, age, occupation, walking time to the nearest health facility, and knowledge level were significantly associated with attitudes toward MMS. In the adjusted model, Knowledge level, pregnancy status, occupation, and walking time to the nearest health facility remained independently associated with positive attitudes.

**Table 3:** Factors associated with positive attitude of multiple micronutrient supplementation among pregnant and lactating women in Rwanda.

Characteristics	Positive attitude *n* (%)	*p-value*	AOR	95% CI	*p-value*
Pregnancy status					
1st and 2nd trimester	109 (51.2)	0.042	1		
3rd trimester	90 (53.2)		1.20	0.77–1.87	0.415
Lactating mothers	75 (69.5)		2.26	1.34–3.80	0.002
Age					
≤ 30	17,353.4	0.007	1		
≥ 30	111 (63.1)		1.25	0.81–1.91	0.299
Marital status					
Living with a partner	146 (56.0)	0.747			
Living with no partner	127 (57.4)				
Education					
None	21 (47.7)	0.451			
Any primary	137 (57.0)				
Any secondary and higher	116 (58.0)				
Occupation					
Agriculture	81 (54.8)	0.004	1		
Business/casual labor	137 (58.4)		0.70	0.43–1.13	0.146
No work	56 (44.8)		0.45	0.26–0.78	0.005
Religion					
Catholic	87 (56.5)	0.943			
Protestant and others	187 (57.0)				
Walking time to the nearest health center					
≤ 30 min	127 (60.0)	0.022	1		
>30 to 60 min	87 (61.3)		0.94	0.58–1.15	0.817
≥60 min	64 (46.7)		0.58	0.36–0.93	0.024
Knowledge level					
Low	169 (47.6)	0.000	1		
High	116 (81.4)		5.09	3.08- 8.43	0.000

AOR, Adjusted Odds Ratio; CI, Confidence Interval. Variables with *p* < 0.20 in bivariate analysis were included in the multivariable logistic regression model.

Knowledge level emerged as the strongest predictor of positive attitudes. Women with high MMS knowledge had substantially higher odds of reporting positive attitudes toward MMS than those with low or moderate knowledge. In addition, lactating mothers were more likely to exhibit positive attitudes than women in the first and second trimesters of pregnancy. Conversely, women with no occupation and those residing farther from health facilities were less likely to report positive attitudes.

The qualitative findings complemented these results by illustrating the perceptions underlying positive attitudes toward MMS. Women frequently associated MMS with improved maternal health, increased blood levels, healthy fetal growth, and reduced risk of anemia. A pregnant woman from Gasabo explained that MMS “*increases blood levels and helps the baby grow well”* (Pregnant Woman, Gasabo District), while a lactating mother from Rutsiro noted that “*these supplements help you give birth to a healthy baby with adequate growth and height”* (Lactating Mother, Rutsiro District). Trust in healthcare providers further reinforced these positive perceptions. As one pregnant woman from Burera stated, “*I trust the health workers because they know what is good for us and our babies”* (Pregnant Woman, Burera District). Participants consistently described healthcare providers as trusted sources of information and guidance on MMS.

Despite these positive perceptions, qualitative findings also identified factors that negatively influenced attitudes toward MMS. Although most participants reported that cultural beliefs did not influence their use of MMS (91%), misconceptions regarding its safety and effectiveness existed. Some women believed that MMS could cause babies to grow excessively large, resulting in difficult deliveries. As one nurse explained, “*Many women fear that MMS will make the baby too large, making delivery more difficult”* (Nurse, Burera District). Healthcare providers also reported that the packaging of MMS contributed to stigma, with some women associating the tablets with HIV medication. One nurse remarked, “*Some women think MMS is for HIV, just because of the way it's packaged”* (Nurse, Burera District).

Participants also described side effects, particularly nausea, vomiting, the unpleasant taste, and the size of the tablets, as factors that discouraged continued use. Nevertheless, many women reported persisting with supplementation because they believed in its health benefits. One participant explained, “*The smell made me want to stop, but I endured because I knew it helped increase blood levels”* (Pregnant Woman, Gasabo District). Household perceptions further shaped attitudes toward MMS. While many husbands supported supplementation, others questioned its safety and effectiveness, and some family members discouraged its use. Participants also expressed differing views on whether MMS offered benefits beyond a healthy diet. Whereas, some questioned the need for supplements when nutritious foods were available, others recognized that MMS complemented dietary intake by providing nutrients that food alone might not adequately supply.

### 3.4 Practice and adherence to multiple micronutrient supplementation

Overall, 92% of participants reported adhering to the recommended MMS regimen (Figure 1). As shown in Table 4, approximately two-thirds of women initiated MMS during the first trimester of pregnancy (66.4%), while nearly one-third started during the second trimester. Evening was the most commonly reported time for taking MMS (45.5%), followed by the morning (35.2%). Overall, 63% of participants reported no barriers to regular MMS use, whereas 37% experienced at least one challenge. Among women reporting barriers, side effects were the most frequently reported concern (93.3%), followed by forgetting to take the supplements (70.5%), while difficulty accessing MMS was uncommon. Most women (87.8%) reported receiving support from their husbands to take MMS, and 91.0% indicated that cultural beliefs did not influence their decision to use the supplements. In addition, 35.7% of participants perceived the taste of MMS as unpleasant, while 62.8% reported that the tablets had an unpleasant smell (Table 4).

**Table 4:** Practices related to multiple micronutrient supplementation among pregnant and lactating women.

Characteristics	Count *N* = 484	%
Time when taking MMS started		
1st trimester	302	66.4
2nd trimester	140	30.8
3rd trimester	16	2.8
Time of day when MMS is taken		
Morning	157	35.2
Noon	17	3.8
Evening	203	45.5
Anytime	69	15.5
Barriers to taking MMS regularly		
No barrier	304	63.0
Any barriers	180	37.0
Reported barriers (*n* = 180)		
Forgetting to take the supplement	127	70.5
Side effects (stomach discomfort, headache, or constipation)	168	93.3
Hard to access	9	5.0
Husband's support in taking MMS		
Support	374	87.8
No support	52	12.2
Cultural beliefs or traditions that influence the decision to take or not take MMS		
Yes	44	9.0
No	440	91.0
Perception of MMS taste		
Good	32	6.7
Bad	173	35.7
No taste	279	57.6
Perception of MMS smell		
Good	53	11.0
Bad	304	62.8
No smell	127	26.2

The qualitative findings complemented these results by highlighting factors that supported regular MMS use. Women frequently described MMS as beneficial for improving maternal health and supporting fetal growth. A pregnant woman from Gasabo explained that MMS “*helps increase blood levels and supports the baby's growth in the womb”* (Pregnant Woman, Gasabo District). Family support and community follow-up also encouraged regular supplementation. As one husband from Rutsiro stated, “*If she forgets to take the supplements, I remind her”* (Husband, Rutsiro District), while a community health worker from Burera noted, “*We visit pregnant women regularly and encourage them to take the supplements as prescribed”* (Community Health Worker, Burera District). Although some women experienced side effects such as nausea, headaches, dizziness, and stomach discomfort, many reported continuing supplementation after receiving advice from healthcare providers on managing these symptoms.

Table 5 presents the bivariate and multivariable analyses of factors associated with MMS adherence. In the bivariate analysis, attitude level, walking time to the nearest health facility, and partner support were associated with adherence and were included in the multivariable model. After adjustment, only attitude toward MMS remained independently associated with adherence. Women with positive attitudes toward MMS had significantly higher odds of adhering to supplementation than those with low or moderate attitudes (AOR = 3.29, 95% CI: 1.42–7.61, *p* = 0.005).

**Table 5:** Factors associated with adherence to multiple micronutrient supplementation among pregnant and lactating women.

Characteristics	Adherent *n* (%)	*p-value*	AOR	95% CI	*p-value*
Knowledge level					
Low/Moderate	297 (90.5)		1		
High	122 (96.0)	0.051	1.78	0.57–5.50	0.315
Attitude level					
Low/Moderate	159 (86.0)	0.000	1		
High	260 (96.3)		3.29	1.42–7.61	0.005
Pregnancy status					
1st and 2nd trimester	176 (92.0)	0.800			
3rd trimester	144 (91.0)				
Lactating mothers	99 (93.4)				
Age					
≤ 30	275 (91.3)	0.423			
≥30	144 (93.5)				
Education					
None	36 (92.3)	0.679			
Any primary	216 (93.1)				
Any Secondary and higher	167 (90.7)				
Occupation					
Agriculture	113 (93.4)	0.441			
Business/casual labor	206 (92.8)				
No work	100 (89.3)				
Religion					
Catholic	137 (92.0)	0.938			
Protestant and others	282 (92.0)				
Walking time to the nearest health center					
≤ 30 min	183 (92.0)	0.092	1		
>30 to 60 min	131 (95.6)		1.89	0.65–5.49	0.237
≥60 min	105 (88.2)		0.75	0.32–1.73	0.504
Partner support					
Yes	334 (93.0)	0.117	1		
No	38 (86.4)		189	0.70–5.11	0.204

AOR, Adjusted Odds Ratio; CI, Confidence Interval. Variables with *p* < 0.20 in bivariate analysis were included in the multivariable logistic regression model.

The qualitative findings support this association, showing that women with positive attitudes consistently expressed confidence in the benefits of MMS and trust in healthcare providers. Although partner support was not independently associated with adherence in the adjusted analysis, interviews suggested that encouragement from husbands and regular follow-up by community health workers reinforced positive attitudes and helped women maintain recommended supplementation practices.

## 4 Discussion

This study provides some of the first evidence on knowledge, attitudes, practices, and adherence to multiple micronutrient supplementation (MMS) during the early phase of program implementation in Rwanda. Although women's knowledge of MMS remained limited, adherence to supplementation was high. Knowledge was the strongest predictor of positive attitudes toward MMS, while positive attitudes emerged as the only independent predictor of adherence. Interviews further demonstrated that perceived benefits of MMS, trust in healthcare providers, family support, and community health worker follow-up reinforced positive attitudes and sustained adherence despite misconceptions, side effects, and limitations in counseling. These findings are consistent with the Health Belief Model, which posits that health behaviors are shaped by perceived benefits, perceived barriers, and cues to action. In the present study, the perceived benefits of MMS encouraged continued supplementation, while misconceptions and concerns about side effects represented perceived barriers to adherence. Trust in healthcare providers, together with support from family members and community health workers, appeared to serve as important cues to action that reinforced women's motivation to continue taking MMS.

Knowledge of MMS remained limited, with only about one-quarter of participants demonstrating high knowledge. Similar findings have been reported in other low- and middle-income countries, where women generally understand how to take supplements but have limited knowledge of their nutritional content and mechanisms of action (16, 18, 20). No socio-demographic characteristics were independently associated with high MMS knowledge, suggesting that knowledge gaps were common across participant groups. Rather than targeting specific population subgroups, these findings indicate that improving the quality of nutrition counseling delivered through routine ANC services may have greater potential to enhance women's understanding of MMS. Although women routinely received information through ANC services and community health workers, counseling often focused on how to take the supplements rather than explaining their composition, purpose, and management of side effects. Healthcare providers attributed these gaps to high client volumes, limited consultation time, inadequate training, and shortages of MMS-specific educational materials. Similar health-system constraints have been shown to compromise the quality of maternal nutrition counseling in other settings (21–23). Together, these findings suggest that the primary challenge lies not in women's access to information but in the quality and comprehensiveness of counseling delivered during routine ANC services.

Women with higher knowledge levels were significantly more likely to report positive attitudes toward MMS, highlighting the importance of effective communication in promoting acceptance of supplementation. Interviews showed that women who understood the benefits of MMS frequently associated it with improved maternal health, increased blood levels, healthy fetal growth, and prevention of anemia. These perceived benefits strengthened confidence in supplementation and fostered favorable attitudes, consistent with previous studies demonstrating that beliefs about the benefits of maternal nutritional interventions are important determinants of positive attitudes and continued supplement use (18, 20, 24, 25). In addition, women residing farther from health facilities were less likely to report positive attitudes, possibly reflecting fewer opportunities for repeated counseling and interaction with healthcare providers. Similar associations between repeated contact with health services, effective counseling, and favorable perceptions of maternal nutrition interventions have been reported in other low-resource settings (18, 25).

The study also identified misconceptions that may undermine positive attitudes toward MMS. Some women questioned whether MMS differed from supplements they had previously received during pregnancy or believed that the tablets contained deworming medicine. Healthcare providers further reported that some women associated the appearance and packaging of MMS with HIV medication, contributing to stigma and reducing confidence in the intervention. Similar uncertainty regarding the identity and purpose of prenatal supplements has been reported during the introduction of MMS in other settings, underscoring the importance of clear communication during programme transition (17, 18, 26). These misconceptions are particularly important because women with greater knowledge were significantly more likely to report positive attitudes. Consistent with the Health Belief Model, inaccurate beliefs may increase perceived barriers while reducing perceived benefits, thereby limiting acceptance of recommended health interventions (15, 24). Conversely, effective counseling can reduce uncertainty, strengthen perceived benefits, and promote more favorable attitudes toward MMS.

Positive attitudes emerged as the strongest determinant of adherence. Women with positive attitudes had more than three times higher odds of adhering to MMS than those with low or moderate attitudes. Interviews suggested that women who believed MMS improved maternal health and fetal growth were more motivated to continue supplementation, even when experiencing temporary side effects. Similar associations between favorable perceptions and adherence to maternal nutrition interventions have been reported in other low- and middle-income countries (20, 27). These findings are consistent with the Health Belief Model, which posits that health behaviors are influenced by perceived benefits, perceived barriers, and cues to action. In the present study, the qualitative findings suggest that women's perceptions of the benefits of MMS encouraged continued use despite temporary side effects, while trust in healthcare providers and regular follow-up by community health workers served as important cues to action that reinforced adherence. Conversely, misconceptions about MMS and concerns about side effects represented perceived barriers that could undermine continued supplementation.

Trust in healthcare providers, family encouragement, and community follow-up further reinforced adherence. Women consistently described nurses, midwives, and community health workers as trusted sources of information whose counseling and reassurance encouraged continued supplement use. Although partner support was not independently associated with adherence in the multivariable analysis, husbands frequently reminded women to take MMS and encouraged ANC attendance, suggesting that family support complemented healthcare provider counseling. Community health workers also reinforced key messages during household visits and addressed concerns regarding supplementation. These findings are consistent with evidence showing that provider communication, family engagement, and community support strengthen adherence to maternal health interventions (5, 18, 25, 28, 29).

Despite the generally high levels of adherence, practical barriers to regular MMS use remained. Women reported treatment-related side effects, forgetfulness, and delayed initiation of ANC as the main challenges to consistent supplementation. In addition, many participants described the taste and smell of MMS as unpleasant. Although these product characteristics did not appear to substantially reduce overall adherence, they may discourage continued use among women experiencing pregnancy-related nausea or discomfort. Similar barriers have been documented in other maternal supplementation programmes and have been associated with delayed initiation and reduced adherence (18, 20, 25). Nevertheless, many women continued taking MMS because they perceived the benefits of supplementation to outweigh the temporary discomfort and had received practical guidance from healthcare providers on managing side effects. These findings highlight the importance of effective counseling and ongoing follow-up in helping women overcome temporary challenges, including unpleasant taste and smell, and maintain adherence throughout pregnancy.

To our knowledge, this is among the first studies to examine knowledge, attitudes, practices, and adherence to MMS during the early phase of implementation in Rwanda using a convergent mixed-methods approach. By integrating quantitative and qualitative findings, the study provides novel implementation evidence on misconceptions related to MMS content and packaging, counseling gaps, provider capacity, and the behavioral, social, and health-system factors influencing adherence. The findings demonstrate that high adherence can be achieved despite limited knowledge when women perceive clear benefits, trust healthcare providers, and receive support from family members and community health workers. The mixed-methods approach enabled triangulation of findings across multiple stakeholder groups, providing a comprehensive understanding of the factors influencing MMS implementation. These findings have important implications for Rwanda's national MMS scale-up, highlighting the need to strengthen ANC counseling through standardized provider training and educational materials, address misconceptions, promote timely ANC attendance, and engage male partners and community health workers to support adherence. The lessons may also inform MMS implementation in other low-resource settings transitioning from iron–folic acid supplementation.

The findings should be interpreted in light of some limitations. First, the cross-sectional design precludes causal inference between knowledge, attitudes, and adherence. Second, adherence was based on self-reported practices and may therefore be subject to recall and social desirability bias. Third, although the knowledge and attitude scales demonstrated acceptable internal consistency (Cronbach's α = 0.60 and 0.65, respectively), the reliability coefficients were modest, suggesting that some measurement error may have remained. Future studies should further validate and refine these instruments. Finally, the study was conducted in three pilot districts and may not fully represent women in districts where MMS has not yet been introduced. Nevertheless, the inclusion of districts with diverse geographical and service-delivery contexts enhances the relevance of the findings for informing Rwanda's national MMS scale-up.

## 5 Conclusions

This study found high self-reported adherence to MMS despite limited knowledge among pregnant and lactating women in Rwanda's pilot districts. Positive attitudes, reinforced by trust in healthcare providers, perceived benefits of MMS, and family and community support, were the strongest drivers of adherence. These findings are consistent with the Health Belief Model, highlighting the importance of reducing perceived barriers and strengthening cues to action to improve adherence to maternal nutritional interventions. Strengthening ANC counseling, addressing misconceptions, promoting timely ANC attendance, and engaging family members, particularly male partners, are essential for improving the implementation of MMS. These findings provide context-specific implementation evidence to inform MMS programmes in Rwanda and other low-resource settings.

## Data Availability

The raw data supporting the conclusions of this article will be made available by the authors, without undue reservation.
