# Supplementary material for: Knowledge, attitudes, practices, and adherence to multiple micronutrient supplementation among pregnant women in Rwanda: a mixed-methods study
Source: Front Nutr. 2026 Aug 28;13:1874824. doi: 10.3389/fnut.2026.1874824 (PMC13561906; doi:10.3389/fnut.2026.1874824)
Supplement: Supplementary file 1 [file Data_Sheet_1.pdf]

Knowledge and attitude scoring procedures. Each knowledge and attitude item was scored dichotomously. Correct responses were assigned a score of 1 and incorrect, "don't know," or unfavorable responses were assigned a score of 0.

Questions	Response options	Score
**Knowledge-related questions**		
1. Which micronutrients do you know are important during pregnancy? (multiple responses)	1= Minerals (iron, calcium, zinc, iodine...)/<br>2= Vitamins (A, D, E, K, C and B)<br>3=Don't know	Any correct answer for either 1 or 2 ==➔ 1 while 3===➔0
2. Which types of micronutrient supplements have you ever heard about?	1=MMS<br>2=IFA<br>3=MNPs<br>4=Do not know	Any correct response on either 1, 3, or 2 ==⇒ 1 while 4===➔0
3. What are multiple micronutrient supplements (MMS)?	1= Tablets containing a combination of vitamins and minerals<br>2= Herbal medicines for general well-being<br>3=Medication for treating specific illnesses during pregnancy<br>4=I don't know	Any correct response corresponding to 1 ==➔ 1 while 2, 4 and 4 ===➔0
4. What micronutrients compose MMS? (MMS contains Vit A, D, E, B3, B9, B1, B2, B6, B12, C, Zinc, Iron, Selenium, Copper, Iodine.) (multiple responses)	1= Vitamins<br>2= Minerals<br>3=Both vitamins and minerals<br>4=I do not know	Any responses 1, 2, and 3 ==➔ 1 while 4 ===➔0
5. When should MMS be taken?	1= Before pregnancy<br>2= During pregnancy<br>3= During breastfeeding<br>4= I don't know	Any responses 1, 2, and 3 ==➔ 1 while 4 ===➔0
6. Why is it important for pregnant women to take MMS? (multiple responses)	1=To prevent anemia<br>2=To improve the baby's development.<br>3= To boost energy levels during pregnancy.<br>4= To treat pregnancy-related complications.<br>5=To prevent many micronutrient deficiencies)<br>6= I do not know.	Any responses 1, 2, and 5 ==➔ 1 while 3 and 4 ===➔0
7. When should a pregnant mother start taking MMS during pregnancy?	1= As soon as you confirm your pregnancy.<br>2=In the second trimester.<br>3=In the last trimester.<br>4=Anytime.	Response 1==➔1 while all 2, 3, 4, 5 ==➔0

	5=I do not know.	
8. How often should MMS be taken?	1= Daily<br>2= Once a week<br>3= Only when feeling tired or weak<br>4= I do not know<br>5=Other	Response 1==➔1 while all 2, 3, 4, 5 ==➔0
9. Can MMS be taken when a mother/woman is not pregnant?	1=Yes<br>0=No<br>2=I do not know	Response 1==➔1 while 0 and 2 ==➔0
10. How many MMS should be taken during pregnancy?	1= 180<br>0= I do not know	Response 1==➔1 and 0==➔0
11. What could happen if a pregnant woman does not take MMS? (multiple responses)	1= She may develop anemia<br>2= Nothing serious will happen<br>3= She will feel fatigued<br>4= Her baby may not grow well<br>5= I do not know.	Response 1, 3, 4, ==➔1 while 0, 2 ==➔0
12. What side effects are associated with taking MMS that you know? (multiple responses)	1= Stomach discomfort<br>2= Constipation<br>3= Headaches<br>4= I don't know.	Response 1, 2, and 3 ==➔1 while 4 ==➔0
**Attitude related questions**		
13. Are micronutrients essential/important during pregnancy?	1=Yes<br>0= No	Response 1 ==➔1 while 0==➔0
14. How important do you think it is for pregnant women to take MMS?	1=Very important<br>2=Somewhat important<br>3=Not important<br>4= I am not sure	Responses 1 and 2 ==➔1 while 3 and 4==➔0
15. How important do you think it is to take MMS supplementation during lactation?	1=Very important<br>2=Somewhat important<br>3=Not important<br>4= I am not sure	Responses 1 and 2 ==➔1 while 3 and 4==➔0
16. What are your main concerns about taking MMS during pregnancy?	1=Possible side effects<br>2=Lack of trust in the supplements<br>3=Difficulty in accessing the supplements<br>4=I have no concerns	Responses 1, 2, and 3 ==➔0 and while 4==➔1

17. Do you think that taking MMS can hurt your baby?/	1=Yes<br>0= No<br>2=Do not know	Response 0 ==➔1 and while 1 and 2 ==➔0. Option 2 was not considered
18. Do you believe that taking MMS can hurt your life?	1=Yes<br>0= No<br>2=Do not know	Response 0 ==➔1 and while 2==➔0. Option 2 was not considered
19. Do you believe taking MMS can help reduce the risk of anemia during pregnancy?	1=Yes, definitely<br>2=I am not sure<br>3=No, it does not help<br>4=I do not know	Response 1 ==➔1 and while 2, and 3 ==➔0. Option 4 was not considered
20. Have you ever been advised by a health professional to take MMS?	1= Yes, regularly.<br>2= Yes, but not frequently.<br>3= No, never.<br>4= I do not remember.	Response 1 ==➔1 and while 2, 3 and 4 ==➔0
21. How much do you trust the advice given by healthcare providers regarding micronutrient supplements?	1=Completely trust<br>2=Mostly trust<br>3=Neutral (either trust or untrust)<br>4=Slightly trust<br>5=Not at all	Responses 1 and 2 ==➔1 and while 3, 4 and 5 ==➔0
22. How satisfied are you with the information provided by healthcare providers about MMS?	1=Very satisfied<br>2=Satisfied<br>3=Unsatisfied<br>4=I have not received any information	Responses 1and 2 ==➔1 and while 3 and 4 ==➔0
23. Would you recommend that other pregnant women take MMS?	1=Yes<br>2=No	Response 1 ==➔1 and while 2 ==➔0
24. Do you feel like you want to stop taking MMS?	1=Yes<br>0=No	Response 1 ==➔0 and while 0 ==➔1

Calculation of composite scores

Composite knowledge and attitude scores were calculated by summing individual item scores (possible range: 0–12 for each domain). Participants scoring **≥6 (≥50% of the maximum score)** were classified as having **high knowledge** or **positive attitudes**, whereas those scoring **<6** were classified as having **low/moderate knowledge** or **less positive attitudes**. Because no validated cutoff exists for this questionnaire, a 50% threshold was used to classify participants into higher and lower knowledge/attitude categories.
